# Direct evidence of an extra-intestinal cycle of *Toxoplasma gondii* in tigers (*Panthera tigris*) by isolation of viable strains

**DOI:** 10.1080/22221751.2019.1682471

**Published:** 2019-10-29

**Authors:** Yurong Yang, Hui Dong, Ruijing Su, Nan Jiang, Tongyi Li, Chunlei Su, Ziguo Yuan, Longxian Zhang

**Affiliations:** a College of Animal Science and Veterinary Medicine, Henan Agricultural University, Zhengzhou, PR People’s Republic of China; b Zhengzhou Zoo, Zhengzhou, PR People’s Republic of China; c Department of Microbiology, University of Tennessee, Knoxville, TN, USA; d Key Laboratory of Zoonosis of Ministry of Agriculture and Rural Affairs, South China Agricultural University, Guangzhou, PR People’s Republic of China

**Keywords:** *Toxoplasma gondii*, extra-intestinal cycle, tiger, isolation, public health

## Abstract

Toxoplasmosis is one of the most common zoonotic diseases in the world. Felines excrete environmentally resistant *Toxoplasma gondii* oocysts. However, there is no direct evidence to prove tigers are the intermediate host of *T. gondii*. Here, we show that, IgG antibodies to *T. gondii* in 80% (8/10) of captive tigers. Two viable *T. gondii* strains (ToxoDB genotype #9) were isolated by bioassay in mice using striated muscles of two tigers (Tiger#3 and Tiger#8). Additionally, mice were confirmed as *T. gondii*-positive by bioassay of feces #89–110, but no viable *T. gondii* strain was isolated successfully. The fecal samples from tigers may contain *T. gondii* oocysts. This is the first report of *T. gondii* isolation from tigers. These results provide direct evidence that an extra-intestinal cycle of *T. gondii* may develop in tigers.

**Dear editor:** Toxoplasmosis is one of the most common zoonotic diseases in the world. Felines specifically excrete environmentally resistant *Toxoplasma gondii* oocysts [1]. The tiger (*Panthera tigris)* was listed as a threatened species by the International Union for Conservation of Nature, as only 3, 200 tigers exist in the world today [2,3]. Captive felids in zoos which are infected with *T. gondii* could be a possible source of contamination for other animals, animal caring staff and visitors. However, there is currently no direct evidence that an extra-intestinal cycle of *T. gondii* occurs in tigers.

In this study, from December 2016 to May 2019, fresh tiger tissues (*n* = 9) or serum samples (*n* = 2) were collected from zoos by local veterinarians and transported to the Laboratory of Veterinary Pathology of Henan Agricultural University for histopathological diagnosis on tissue samples, serological diagnosis on serum and heart juice samples. Fresh fecal samples (*n* = 141) from 32 individuals (four to five samples per tiger) were also collected from the individual living houses of tigers in the morning by the authors of this study (Table 1, Figure S1). The food of captive tigers included raw beef, pork, and chicken ribs. Histopathological diagnosis revealed that pneumonia, declined immune function, and reproductive disorders were the common causes of tigers death in the majority of cases (Figure S2).

**Table 1.** Background and isolation of *Toxoplasma gondii* from tigers.

Samples (*T. gondii* ID)	City	Sample received date	Subspecies	Age (month, sex)	Clinical symptoms, pathology finding	MAT	Mice bioassay ^d^	
							BALB/C	γ-IFN^-/-^
Tiger #1^a^	Kaifeng	Dec 30, 2016	*Panthera tigris tigris*	≥120, M	Depressed, anorexia. Spleen, liver and kidney were atrophied.	1:50	0/5	nd
Tiger #2	Zhengzhou	Feb 14, 2017	*Panthera tigris altaica*	6, F	Only serum.	1:400	nd	nd
Tiger #3^b^ (TgTigerCHn1)		May 29, 2017		6, F	Bloody diarrhea, anorexia, tachypnea and salivation.	≥1:200^c^	3/4	2/2
Tiger #4		Jul 21, 2017		1, M	Artificial feeding, history of fracture, anorexia, and pulmonary congestion. Spleen, liver, and kidney were immature.	≥1:200^c^	0/2	nd
Tiger #5		Oct 18, 2017		0, M	Stillborn fetus.	<1:25	nd	nd
Tiger #6		Dec 8, 2017		≥180, F	Dystocia (2 weeks before death), uterus was ruptured, peritonitis, and sepsis.	≥1:200^c^	0/2	nd
Tiger #7		Dec 12, 2017		2, M	Liver congestion and swelling, intestine synechia to the abdominal wall, and spleen swelling. Frozen tissues.	≥1:200^c^	nd	nd
Tiger #8 (TgTigerCHn2)		Dec 18, 2017		48, M	Weight loss, right forelimb could not touch the ground, and mouth twitching. Spleen and lymphonodus necrosis, and interstitial pneumonia.	≥1:200^c^	1/5	2/2
Tiger #9		Oct 25, 2018		300, M	Old, anorexia, multiple organ failure, and atrophy.	1:400	0/5	0/2
Tiger#10	Luoyang	May 14, 2019		9, M	Artificial feeding and abnormal nervous symptoms. Frozen tissues.	<1:25	nd	nd
Feces #1–36	Zhengzhou	Oct 31–Nov 6, 2016	*Panthera tigris altaica*	–	–	–	0/5	nd
Feces #37–69		Nov 8–Nov 19, 2016		–	–	–	0/5	nd
Feces #70–88		Dec 5–Dec 10, 2016		–	–	–	0/4	nd
Feces #89–110		Feb 29–Mar 15, 2017		–	–	–	1/3	nd
Feces #111–141	Shangqiu, Puyang	July 19–Aug 16, 2017		–	–	–	0/5	nd

^a^*T. gondii* DNA amplified products were found in the heart, diaphragm and skeletal muscles of Tiger #1.

^b^*T. gondii* DNA amplified products were found in the heart, tongue and skeletal muscles digestion liquids of Tiger #3.

^c^End titration not performed.

^d^Number of positive mice/number of inoculated mice.

nd: Experiment not done.

In order to investigate *T. gondii* infection in these tigers, *T. gondii* antibodies were identified by the modified agglutination test (MAT) (cut off = 1:25) [4]. Results revealed that *T. gondii* IgG antibodies were found in the heart juice or serum of eight tigers (80%) (Table 1). This indicated that most of the tigers had been infected with *T. gondii*. The tigers may have been infected with *T. gondii* after ingesting viable cysts from raw meat or oocysts from contaminated food and water. *T. gondii* antibodies were negative in Tiger #5 (stillborn fetus) and Tiger #10 (artificial feeding, 9 months). *T. gondii* was previously isolated from captive meerkats, and it was thereby speculated that the oocysts shed by captive felids or feral cats contaminated the zoo environment [5].

DNA was extracted by silica membrane from the tissue samples and used to detect *T. gondii* by PCR using primer Tox5/8 [6]. *T. gondii* DNA amplified products were found in the heart, tongue, diaphragm, and skeletal muscles of Tigers #1 and #3 (Table 1). Striated tissue from six *T. gondii* seropositive tigers were subjected to acid pepsin digestion and bioassayed in mice [1]. The other three samples were stillborn fetuses or frozen tissues. Two viable *T. gondii* strains were obtained from seropositive tiger samples (MAT titer ≥ 1:200), this result verified the validity of MAT use on tiger samples. *T. gondii* antibodies and parasites were detected in mice inoculated with tissues from Tigers #3 and #8 at 61 days post-infection (DPI). Additionally, many brain tissue cysts were observed in mice inoculated with tissues from Tigers #3 (223 ± 224, 249 DPI) and #8 (7640 ± 824, 102 DPI) after euthanasia. The parasites were confirmed to be *T. gondii* by immunohistochemical staining (Figure 1). IFN-γ knockout mice died of toxoplasmosis at 14 and 8 DPI after inoculation with samples from Tigers #3 and #8, respectively, and tachyzoites were found in smears of the lungs, mesenteric lymph nodes, and ascites. The two isolates were successfully propagated in *Vero* cells, TgTigerCHn1 and TgTigerCHn2 (Table 1). DNA samples extracted from *T. gondii* tachyzoites in cell cultures were characterized by PCR-RFLP [7]. They were identified as ToxoDB#9, the predominant genotype found in China [8,9]. The ROP18/ROP5 genotype combination (II/II) predicated that they were non-lethal to mice [10]. However, TgTigerCHn1 and TgTigerCHn2 were found to be of intermediate virulence and virulence to mice, respectively (Table S1). This result might indicate that there are still other factors related to virulence.

**Figure 1.**
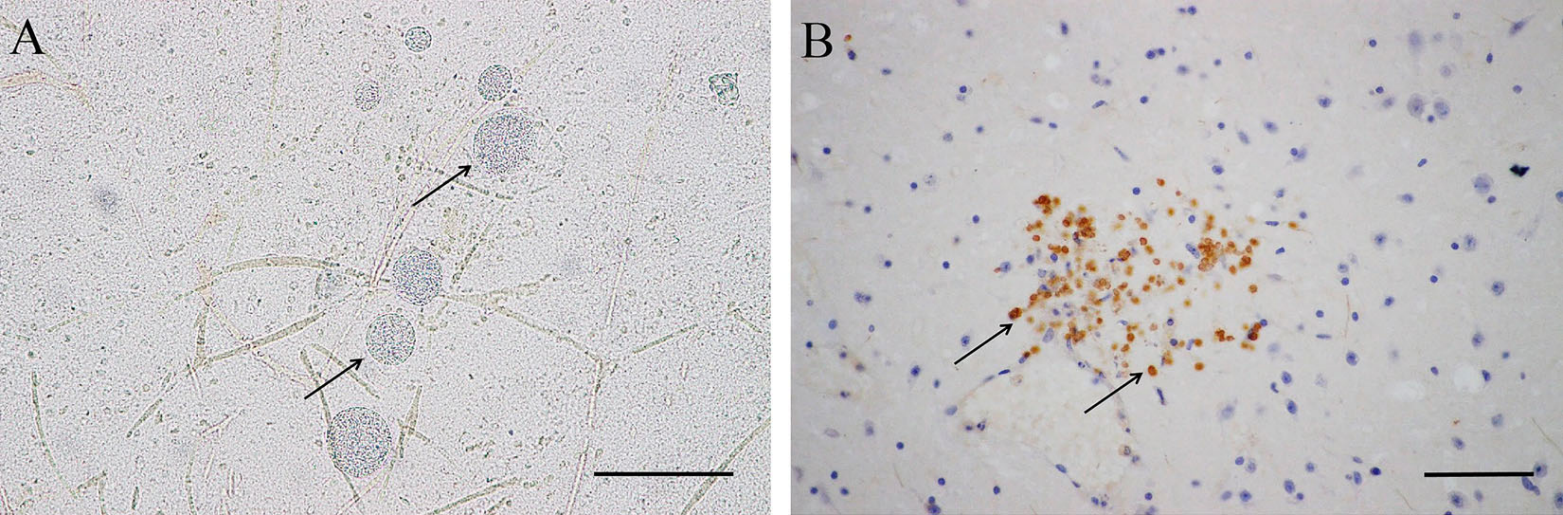
Morphology of *T. gondii* cysts and tachyzoites in brains of BALB/C mice. A. *T. gondii* cysts (TgTigerCHn1), 61 DPI, squash, unstained. B. *T. gondii* tachyzoites (TgTigerCHn2), 10 DPI, IHC stained (rabbit anti-*T. gondii* antibody). Bar = 50 μm.

The 141 fecal samples were divided into five groups. A bioassay was conducted on BALB/c mice. One group of mice (pool feces #89–110) was *T. gondii* positive (MAT ≥ 1:200). Unfortunately, this strain was not isolated successfully (Table 1). In the natural environment, felids shed *T. gondii* oocysts for a short period of time, and oocysts were found in only 1% of cats at any given time, according to fecal surveys conducted from 1988–2008 [1]. Seropositive samples gave negative results in mice, which may be explained by the relatively low density, low cyst formation rate, or avirulence of *T. gondii*. Little is known about the isolation of viable *T. gondii* from the feces of feral felids. Two *T. gondii* strains were previously isolated from the feces of cougars [11]. Inactivating (burn or heat) oocysts and cleaning the feces from captive felids are important strategies for controlling *T. gondii* infection.

To our knowledge, this is the first report of *T. gondii* isolation in tigers. The tigers in this study were captive and bred in zoos. Tiger blood samples were not collected at the time of capture and were not checked for *T. gondii*. The infection source of *T. gondii* may be from the meat provided by the zoos or after the ingestion of oocysts from felid feces. Eating pre-frozen meat to break down the transmission route may be the most effective method for preventing infection. These results provide direct evidence that an extra-intestinal cycle of *T. gondii* occurs in tigers.

## Supplementary Material

Supplemental Material
