# Supplementary material for: Direct evidence of an extra-intestinal cycle of Toxoplasma gondii in tigers (Panthera tigris) by isolation of viable strains
Source: Emerg Microbes Infect. 2019 Oct 29;8(1):1550–2. doi: 10.1080/22221751.2019.1682471 (PMC6830256; doi:10.1080/22221751.2019.1682471)
Supplement: Supplemental Material [file TEMI_A_1682471_SM2594.doc]

**Supplementary Figures and table**

**
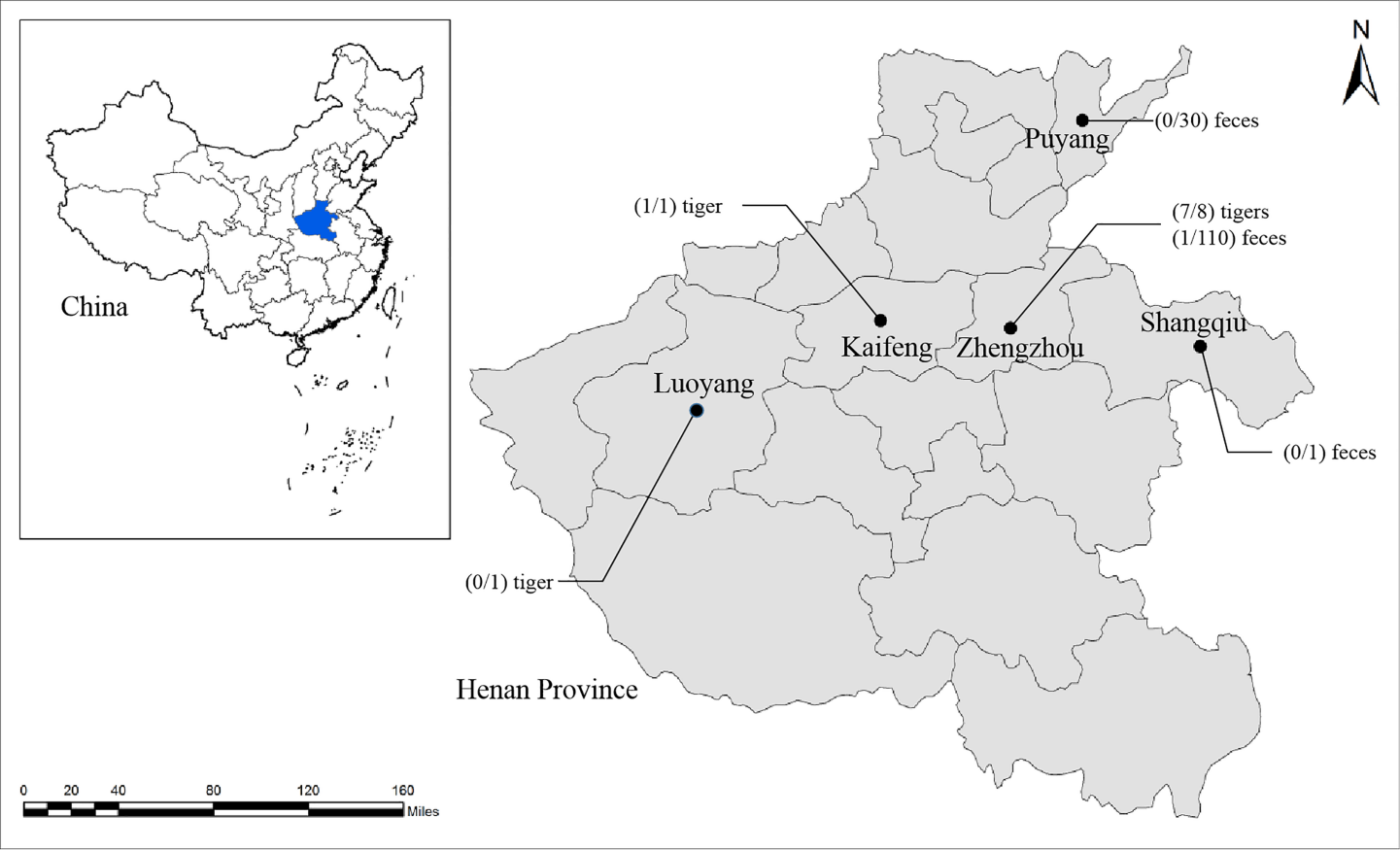
**

**Figure S1. Location of tiger samples received from Henan Province, China.**

MAT results of *Toxoplasma gondii* antibodies of tiger meat juice, and results of the feces bioassay in mice.

Map was adapted from Wikipedia.

Note: No. positive samples/No. test samples.

**
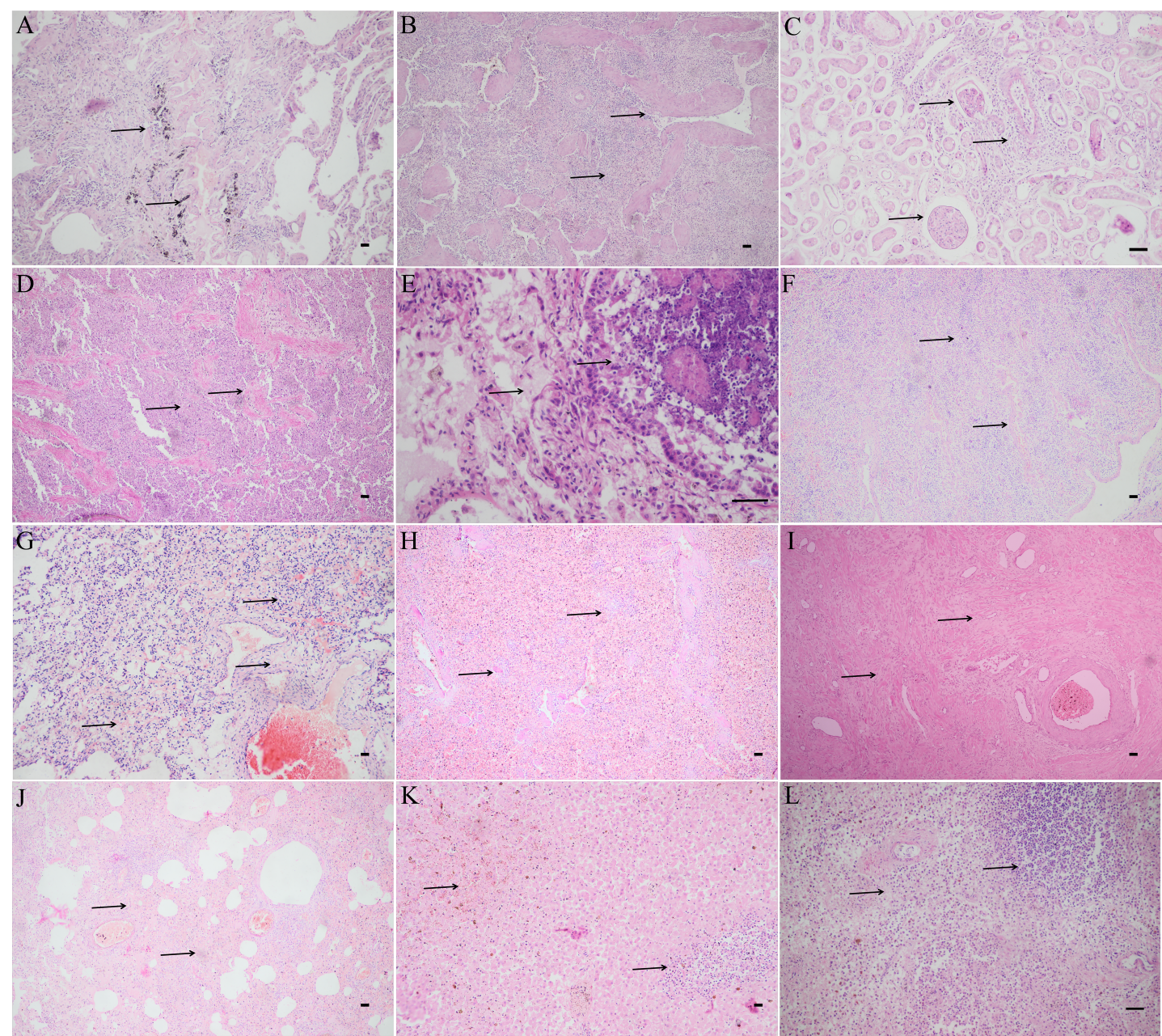
**

**Figure S2. Pathological findings from tiger samples.**

1. Black foci are peribronchiolar deposits of dust, lungs, Tiger #1. B. Spleen was atrophied, trabecula hyperplasia, Tiger #1. C. Monocyte cells infiltrated the mesenchyme, glomerulus was atrophied, Tiger #1. D. Diffuse necrosis of the spleen, Tiger #3. E. Pulmonary edema and suppurative bronchitis, Tiger #3. F. Spleen was atrophied, trabecula hyperplasia, Tiger #3. G. Monocytes and fibrin infiltrated the alveolus, lung, Tiger #4. H. Diffuse necrosis, hemosiderin present in the spleen, Tiger #6. I. Necrosis of the myometrium, uterus, Tiger #6. J. Interstitial pneumonia, monocytes and lymphocytes were infiltrated, Tiger #8. K. Focal hemorrhage and necrosis of the liver, Tiger #8. L. Necrosis of the spleen, Tiger #8. Bar = 50 µm, H&E stained.

**Table S1. Pathogenicity of *T. gondii* strains isolated from tiger samples in BALB/C mice by intraperitoneal inoculation (60DPI)**

| No. of tachyzoites | 10^0^ | 10^1^ | 10^2^ | 10^3^ | 10^4^ | 10^5^ | 10^6^ |
| --- | --- | --- | --- | --- | --- | --- | --- |
| **TgTigerCHn1** | | | | | | | |
| *T. gondii* positive rate  (No. infected/ No. inoculation) | 20%  (1/5) | 80%  (4/5) | 100%  (5/5) | 100%  (5/5) | 100%  (5/5) | 100%  (5/5) | 100%  (5/5) |
| No. dead in acute infection/No. infected | 0/1 | 2/4 | 1/5 | 3/5 | 3/5 | 5/5 | 5/5 |
| Median survival days | ≥60.0 | 24.5 | 26.0 | 17.0 | 18.0 | 14.0 | 13.0 |
| **TgTigerCHn2** | | | | | | | |
| *T. gondii* positive rate  (No. infected/ No. inoculation) | 20%  (1/5) | 20%  (1/5) | 80%  (4/5) | 100%  (5/5) | 100%  (5/5) | 100%  (5/5) | 100%  (5/5) |
| No. dead in acute infection/ No. infected | 1/1 | 1/1 | 4/4 | 5/5 | 4/5 | 5/5 | 5/5 |
| Median survival days | 17.0 | 17.0 | 13.5 | 13.0 | 12.0 | 10.0 | 9.0 |
| **Log-rank (Mantel-Cox) test** | | | | | | | |
| Hazard ratio | 0.1353 | 0.0498 | 0.1770 | 0.3998 | 0.7055 | 0.0814 | 0.1111 |
| *P -* value | 0.3173 | 0.1573 | 0.1439 | 0.3983 | 0.8292 | 0.0086* | 0.0162* |

**p* < 0.05.
